# Trocar Entry Site Hernias in Laparoscopic Sleeve Gastrectomy Patients: A Retrospective Cross-Sectional Study

**DOI:** 10.7759/cureus.49538

**Published:** 2023-11-28

**Authors:** Medeni Şermet

**Affiliations:** 1 General Surgery, İstanbul Medeniyet University, Goztepe Prof. Dr. Suleyman Yalcin City Hospital, Istanbul, TUR

**Keywords:** hernia, bariatric, gastric sleeve, trocar site hernia, unrepaired fascia

## Abstract

Introduction

There is insufficient data regarding trocar access site hernias (TSH) in laparoscopic sleeve gastrectomy (LSG). This retrospective study aimed to identify the incidence and risk factors for hernia development in patients who did not undergo fascia repair at trocar entry sites.

Materials and methods

We retrospectively reviewed the records of 284 patients with morbid obesity who underwent LSG between January 2016 and December 2021. The fascia of the trocar entry site was not closed in any of the patients. Weight, body mass index (BMI), percentage of excess weight loss (%EWL), percentage of total weight loss (%TWL), comorbidities, and the occurrence of complications were recorded at one, six, 12, 18, and 24 months after surgery. Ultrasonography (USG) was performed and supplemented with computed tomography (CT) when necessary.

Results

All patients underwent a 24-month follow-up, during which four patients developed trocar site hernias, resulting in an overall prevalence of 1.4%. Of the total hernias, two occurred within the first 30 days. A single patient required surgical intervention for an incarcerated hernia on the 18th day. Before undergoing laparoscopic sleeve gastrectomy (LSG), the mean weight and body mass index (BMI) of the participants were 124.2 ± 16.7 kg and 43.4 ± 5.7 kg/m², respectively. After one year, the participants experienced a mean percentage of excess weight loss (EWL) of 77.1 ± 12.2% and a mean total weight loss (TWL) of 33.2 ± 6.2%. Hernia formation has been found to be associated with both type 2 diabetes (T2D) and female gender.

Conclusion

In laparoscopic sleeve gastrectomy (LSG), repair of the trocar port closure is not always necessary. The rates of hernia at port entry sites were similar between cases with and without fascial repair.

## Introduction

Trocar or port site hernia (TSH) is an incisional hernia that occurs at the trocar site after minimally invasive surgery [1]. Early diagnosis is essential to prevent the serious morbidity and mortality associated with TSH. The frequent use of laparoscopic surgery for many abdominal procedures has led to an increase in the incidence of TSHs (1%-6%) and associated complications [2,3]. Bariatric surgery involves two significant risk factors that contribute to hernia formation: obesity and challenges in closing the entry site due to inadequate visualization of the fascia in small deep wounds. The closure of all layers from the fascia to the skin at the trocar site using the standard technique poses a significant challenge. Several techniques have been suggested to close trocar wounds while minimizing the risk of hernia formation, including the use of specialized devices, endoscopic suturing, or different trocar insertion methods. However, these options are costly in terms of time or money [4]. We present our experience at a single center where trocar incisions were not repaired. Our objective was to identify hernia formation at trocar insertion sites without the use of fascia repair during the 24-month follow-up period, which could offer evidence to determine the necessity of fascia repair.

## Materials and methods

This retrospective study utilized data from patients who underwent laparoscopic sleeve gastrectomy (LSG) at our clinic between January 2016 and December 2021. Patient information, operation details, postoperative complications, and routine follow-up data were recorded during this period in computerized patient follow-up files. Following our usual follow-up protocol, all patients underwent superficial ultrasonography (USG) at trocar entry sites at six, 12, and 24 months, without any associated complaints. Data collected from these scans were retrospectively analyzed to investigate the frequency of trocar site hernias.

Surgical procedure

All surgeries were conducted laparoscopically with the use of four or five trocars. In every instance, insufflation up to 14 mmHg was administered to establish pneumoperitoneum by utilizing a 10 mm reusable trocar positioned 3-4 cm above the umbilicus to the upper left. The trocars utilized were bladed and included two 5 mm trocars, one 10 mm trocar, and one 15 mm trocar for the LSG. In some instances, an additional 5 mm port was inserted as the fifth port owing to omental size, adhesions, and visualization issues. The trocars were removed by visualization at the conclusion of each procedure. The port sites that underwent LSG were not subjected to fascia repair.

Follow-up

Patients received standard postoperative follow-up, including clinic visits at one-week, one-month, three-month, six-month, 12-month, 18-month, and 24-month intervals.

During each visit, data on weight, body mass index (BMI), percentage of excess weight loss (%EWL), and percentage of total weight loss (%TWL) were collected. Ultrasonography (USG) was used to check the trocar site for hernia onset at one, six, 12, and 24 months, while computed tomography (CT) was used in suspicious cases.

Statistical analysis

The results are reported as either the median or the mean ± standard deviation (SD) for continuous variables and as the number (percentage) for categorical variables. Analyses were conducted using Statistical Package for Social Sciences (SPSS) software version 19.0 for Windows (SPSS, Chicago, IL).

## Results

Over a span of six years, bariatric surgery was performed on 321 patients diagnosed with obesity. Patients who preferred procedures other than LSG due to differences in trocar access and patients with insufficient follow-up data (n=37) were excluded from this study. Therefore, 284 patients were included in this study. The mean age of the participants was 36.9 years, with a mean weight and BMI of 124.2 ± 16.7 kg (range: 95-192 kg) and 43.4 ± 5.7 kg/m² (range: 37.7-70.2 kg/m²), respectively (Table 1).

**Table 1:** Patient demographics and clinical characteristic features SD: standard deviation, BMI: body mass index, EBW: excess body weight, OSAS: obstructive sleep apnea syndrome, T2D: type 2 diabetes

Parameters	Study patients (n=284) (mean ± SD)	Range
Female	237 (83.5%)	18-64
Male	47 (16.5%)	21-61
Age (years)	36.9 ± 1.34	18-64
Weight (kg)	124.2 ± 16.7	96-192
BMI (kg/m^2^)	43.4 ± 5.7	37.7-70.2
EBW (kg)	63.4 ± 4.46	37.3-124.1
OSAS	14 (4.9%)	-
T2D	96 (33.8%)	-
Hypertension	72 (25.3%)	-
Dyslipidemia	84 (29.5%)	-

Data from 284 patients were available for review. After a mean follow-up period of 24 months, four hernias were detected in four patients (Table 2).

**Table 2:** TSH development according to the number of trocars entered TSH: trocar site hernia

Time (months)	5 mm (n=655)	10 mm (n=284)	15 mm (n=284)
0-1	0	1	1
1-6	0	0	0
6-12	0	1	1
12-18	0	0	0
18-24	0	0	0

The incidence rate of trocar site hernia was 1.4% (4/284). Our data revealed two hernias in the right hypochondrium (15 mm wound with a knife trocar) and two hernias in the left upper lateral umbilicus (10 mm trocar). No herniation was found in the 5 mm wounds. No mortality was observed during the follow-up period. Among these hernias, we performed segmental jejunal resection and side-to-side anastomosis in a 10 mm incarcerated Richter's hernia that developed within the first month due to necrosis. The medical records of patients who presented with trocar site hernia showed that three had T2D, whereas one had no identifiable risk factors. Postoperative weight and BMI averaged 102.3 ± 17.1 kg (range: 82.1-151 kg) and 43.8 ± 5.7 kg/m² (range: 34.5-47.4 kg/m²), respectively. The mean percentage of excess weight loss (%EWL) was 70.6% (Table 3).

**Table 3:** Characteristics of patients with hernia %EWL: percent excess weight loss, %TWL: percent total weight loss, USG: ultrasonography, CT: computed tomography, BMI: body mass index, EBW: excess body weight, TSH: trocar site hernia

	Patient 1	Patient 2	Patient 3	Patient 4
Age/gender	27/female	38/female	43/female	46/female
Comorbidity	-	T2D	T2D	T2D
BMI (kg/m²)	47.8	48.4	43.3	45.8
EBW (kg)	64.3	54.7	51.3	52.9
%EWL	-	-	-	-
%TWL	-	82.3	-	91.5
Trocar size	10 mm	15 mm	10 mm	15 mm
Time to develop TSH	18 days	6-12 months	15 days	6-12 months
Symptom	Abdominal pain and vomiting	-	Swelling at the trocar site	-
USG	1	1	1	1
CT	1	-	1	-
Treatment	Segmentary jejunum resection	Follow-up	Follow-up	Follow-up

## Discussion

In this study, we examined the records of 284 consecutive patients to assess the incidence of TSH during LSG procedures performed without closure of the trocar access site fascia. Additionally, we identified predisposing factors for TSH development and determined that our series had a TSH level of 1.40%. Among the four patients who experienced hernias, the port diameter ranged from 10 to 15 mm; two hernias developed within the first month, while the other two emerged between six and 12 months. The risk factors for hernia development include T2D and female sex.

In 1994, the first large-scale study of TSH in gynecologic cases was published, and the incidence of TSH was reported as 0.021%. There were more than four million cases in this study, and 82.1% of cases with TSH developed in trocars over 10 mm. Of the hernias, 75.7% developed in the umbilical region [5].

Regarding other postoperative outcomes, one study showed that nonfacial closure can provide multiple advantages. Closure defects generally require additional time and may prolong operation time [6]. In a comprehensive systematic review of 22 articles, a mean pooled estimate of a 0.5% incidence of TSH after laparoscopic surgery was identified, with a range of 0%-5.2% [7]. Long-term follow-up may reveal an asymptomatic population, but not all patients require treatment. Therefore, it is worth conducting a cost-effectiveness analysis to determine whether an extended follow-up procedure is necessary.

Numerous risk factors related to TSH have been identified, which are dependent on both patient characteristics and surgical techniques. Notable factors that increase susceptibility in obese patients include higher intra-abdominal pressure, thicker peritoneum, and a larger preperitoneal space that guarantees full-thickness closure [8]. Studies have also suggested that obesity alone may be a contributing factor to incisional hernia, as it increases the risk of wound infections [9]. Furthermore, trocar hernia formation is influenced by advanced age, sex (with males being more susceptible than females), nutritional status, diabetes, anemia, steroid therapy, renal failure, cancer, and wound infections. The risk factors associated with surgery include prolonged operative time, excessive handling of the trocar site, the size of the trocar incision, the location of the trocar insertion (umbilical areas are more susceptible to hernias), fascial failure to close the trocar, and the location and type of the trocar tip (bladed, bladeless, or radially expanding) [10-12]. However, not all of these risk factors have been conclusive.

In our study, all patients diagnosed with hernia were female, which differs from what is presented in the current literature. We attribute this trend to the fact that 83.5% of our patients were female. Additionally, T2D stands out as a risk factor for herniation, which we believe contributes to the delayed wound healing.

Some scholars suggest that trocar position is more critical than size or risk factors [13-15]. Consequently, variations in laparoscopic trocar tip design have sought to minimize the occurrence of hernias.

A particular study observed that a bladeless trocar enabled tissue penetration without severing abdominal muscle fibers, resulting in reduced bleeding at the trocar incision point and lower overall complications [16]. However, some laparoscopic instruments require larger trocars. Controversy surrounds the routine closure of fascial defects.

In our practice, we insert a 10 mm trocar at a slight angle approximately 3 cm proximal to the umbilicus, passing through the rectus muscle on the left lateral side. This angled entry strategy guarantees complete closure of the defect after the removal of the trocar. Conversely, a 15 mm trocar was inserted at a slight angle approximately 5 cm beneath the right subcostal area. Typically, there are no mobile organs in this region, which inhibits herniation in this area upon trocar exit. As this could have detrimental effects, several studies have suggested closing the defect when using a 10 mm trocar [17,18]. Prior data on other laparoscopic procedures have established the safety and practicability of leaving the fascia open following the use of a 10 mm trocar [17]. Furthermore, a separate study discovered a correlation between the prevalence of TSH and drain positioning via trocar ports [19]. It is possible that the hernia formation found at the location of the 15 mm trocar in our investigation was associated with the placement of the drain through this port [20,21].

TSH can be asymptomatic or manifest as nausea, vomiting, abdominal pain, fatigue, or swelling. Hernia-related symptoms may initiate pain and lead to bowel necrosis. Although the timing of these symptoms can range from a few days to several months post-intervention, they generally commence within days after surgery [22,23]. Our data revealed two hernias within the first 30 days, and one patient experienced incarceration. Subsequent hernia development occurred after the wound-healing process was complete. Based on the fact that all patients underwent intermittent ultrasonography, it can be concluded that leaving the fascia open is comparable to closing it in the event of hernia development. To diagnose potential hernias, abdominal CT and ultrasonography can be used in conjunction with symptoms.

Among the risk factors for TSH in LSG, T2D is significant in our study. However, we were unable to establish a connection between fast weight loss and hernia formation, particularly during periodic follow-up within the first year. Nonetheless, we attribute the development of TSH in some patients to heavy workload, constipation, uncontrolled strenuous exercise, and sudden movements during the recovery period. From an alternative viewpoint, TSH may not be attributed to a solitary factor but instead to the amalgamation of numerous factors, and the weakening of the abdominal wall caused by unnoticed small vessel damage is a major risk factor for TSH. Therefore, determining a safe insertion technique for trocars to prevent vascular injury is crucial to reduce the risk of TSH. Although surgical communities have adopted different techniques, the correct access route for all patients remains unclear.

Once TSH occurs, it cannot heal spontaneously, and surgical repair is the only treatment. However, most asymptomatic cases require follow-up observations only.

Our study has some limitations. First, its retrospective design limited the reliability of the study. Second, it was not compared with a group that underwent fascial closure. However, the strengths of our study include the regular follow-up of all cases using USG.

## Conclusions

Repair of trocar access sites is not mandatory in LSG operations, because results similar to those in the literature have been obtained. Trocar ports should be selected outside the umbilicus to reduce the risk of herniation. Manipulations to enlarge port sites should be avoided, and some port sites should be repaired based on personal experience. Large-scale studies on TSH levels in bariatric surgery procedures, especially LSG, are needed.
